# Not Getting What You Want? The Impact of Income Comparisons on Subjective Well-Being—Findings of a Population-Based Longitudinal Study in Germany

**DOI:** 10.3390/ijerph16152655

**Published:** 2019-07-25

**Authors:** André Hajek, Hans-Helmut König

**Affiliations:** Department of Health Economics and Health Services Research, University Medical Center Hamburg-Eppendorf, 20246 Hamburg, Germany

**Keywords:** income comparisons, longitudinal study, subjective well-being, life satisfaction, emotions, GSOEP, relative income, happiness

## Abstract

Previous studies have mainly focused on interindividual income comparisons (e.g., comparisons with colleagues or neighbors), whereas intraindividual income comparisons (i.e., difference between factual income and expectations) have rarely been investigated in well-being research. Thus, the aim of this study was to investigate the role of intraindividual income comparisons on subjective well-being (negative/positive emotions and life satisfaction) longitudinally. Data from 2005 to 2013 (biannually) were used from the German Socio-Economic Panel (GSOEP), a nationally representative, longitudinal study. Affective well-being (negative and positive emotions) were quantified by using the affective well-being scale-SOEP. Life satisfaction was quantified using the widely used one-item form. Intraindividual income comparisons were analyzed by using the distance between the own individual income and fair income (“how high would your net income have to be in order to be just”). We tested whether negative (i.e., factual income was lower than their self-rated just income) and positive income comparisons (otherwise) affect the outcome measures differently. Fixed effects regressions showed that positive emotions increased with positive income comparisons in the total sample (*β* = 0.16, *p* < 0.05). In contrast, negative income comparisons neither affect negative emotions nor satisfaction with life. Strategies to shift income expectations might be beneficial for increasing positive emotions.

## 1. Introduction

More than 30 years ago, Richard Easterlin published a famous study about the relation of subjective well-being (SWB) and the growth of monetary factors [1]. He stated that richer individuals were more satisfied with their lives than poorer individuals were, cross-sectionally. Paradoxically, monetary growth over time does not increase satisfaction with life in industrialized countries longitudinally. This paradox is also known as “Easterlin-Paradox”. An often-used explanation is that income matters in relative terms rather than in absolute terms, i.e., the SWB of individuals depends on income comparisons (own income in comparison to a given reference group such as neighbors or colleagues [2], rather than absolute income (such as own individual net income).

Furthermore, there are some asymmetries in income comparisons. It was often found that satisfaction with life is lower when an individual’s income is lower than the income of a reference group (negative income comparisons). However, individuals with an income above a given reference group, usually do not report a better SWB. Consequently, it was concluded that income comparisons are often upwards [3,4].

Yet, most of the previous studies focused on life satisfaction (cognitive evaluation of life as a whole, also known as “cognitive well-being”) as well as happiness (please see the discussion section for further details). However, SWB mainly consists of life satisfaction and affective well-being covering positive (e.g., happiness) as well as negative emotions (anger, worries or sadness) [5]. Moreover, which is worth underlining, most of the previous studies focused on interindividual income comparisons (for example, income comparisons with colleagues or neighbors), whereas intraindividual income comparisons (i.e., difference between factual income and expectations) have rarely been investigated longitudinally. For example, a very recent study investigated the impact of perceived income injustice on stress-associated diseases (e.g., diabetes, stroke) [6]. They found that an income perceived as unjust for over five years was associated with stress-related diseases in women and men.

In sum, the purpose of the present study was to examine whether negative and positive income comparisons affect SWB differently (asymmetric effects). Hence, we provide first insights into the link between intraindividual income comparisons and SWB. Moreover, it is important to explore whether the concept of interindividual income comparisons—which was examined in several existing studies [3,7]—can be extended to intraindividual income comparisons. Additionally, this knowledge might be crucial to create interventions (shifting income expectations) to maintain well-being. This in turn, is important because SWB is known to contribute to health and longevity [8].

## 2. Materials and Methods

### 2.1. Sample

Data were gathered from the German Socio-Economic Panel (GSOEP), which is administered by the German Institute for Economic Research, DIW Berlin. Starting in 1984, the GSOEP is an annual, interdisciplinary (topics such as income, employment status, health, social exclusion or satisfaction), longitudinal, population-based study in Germany. At the micro-level, changes over time in individuals or households can be analyzed. About 11,000 households and over 20,000 individuals (Germans living in the Old and New German States, Foreigners, and recent Immigrants to Germany) were interviewed every 12 months. Very high wave-to-wave reinterview response rates [9] and low survey attrition rates [10] were consistently reported for the GSOEP. Further details regarding the sampling frame and the sample composition is provided elsewhere [11].

In the present study, for reasons of data availability (please see the sections ‘Dependent Variables’ and ‘Intraindividual Income Comparisons’ for further details), the waves:

- (a) 2005, 2007, 2009, 2011 and 2013 were used with life satisfaction as outcome measure in regression analysis.
- (b) 2007, 2009, 2011 and 2013 were used with negative or positive emotions as outcome measure in regression analysis.

Participants gave their informed consent prior to data collection. An ethical approval was not obtained because criteria for the need of an ethical statement were not met (risk for the respondents, lack of information about the aims of the study, examination of patients). However, the German Council of Science and Humanities (Wissenschaftsrat) evaluated the German Socio-Economic Panel at the Deutsches Institut für Wirtschaftsforschung (DIW), Berlin, Germany. The German Council of Science and Humanities approved the GSOEP. The GSOEP is in accordance with the Helsinki Declaration as revised in 2008.

### 2.2. Dependent Variables

Schimmack and colleagues [12] developed the affective well-being SOEP (AWB-S) scale in order to quantify affective well-being, consisting of four items. From 2007 onwards, participants were asked about their affective well-being. Participants indicated for each feeling how often or rarely they experienced this feeling in the last four weeks. Thus, they were asked: “How often have you felt” … (i) angry, (ii) worried, (iii) happy, and (iv) sad? (1 = “very rarely”, 2 = “rarely”, 3 = “occasionally”, 4 = “often”, and 5 = “very often”). We focused on both negative (anger, worries, or sadness) and positive emotions (happiness). To this end, an index score was generated for negative emotions by averaging the score of the corresponding items (from 1 = rare negative emotions, to 5 = frequent negative emotions). To put it another way: The higher the score, the more frequent the negative emotions. The same held true for positive emotions. It was shown that the AWB-S has satisfactory psychometric properties [12].

Life satisfaction was quantified using the widely used one-item form (ranging from 0 = “completely dissatisfied”, to 10 = “completely satisfied”). The reliability of single-item life satisfaction measures has been demonstrated [13].

### 2.3. Independent Variables

From 2005 onwards, individuals were asked every second year “Is the income that you earn at your current job just, from your point of view?” (yes; no). If they answered “no”, they were specifically asked “how high would your net income have to be in order to be just” (denoted as y_r_). The factual individual net income (last month) was denoted as y.

In order to test for asymmetric effects, we created variables “poorer” and “richer” (see Table 1) as proposed by Ferrer-i-Carbonell (please see Ferrer-i-Carbonell for further details [3]): 

Thus, individuals were classified as “richer” (“poorer”) if their factual income (y) was higher (lower) than their self-rated just income (y_r_). As suggested by Ferrer-i-Carbonell [3], among others, the difference between the logarithm of the individual’s own income and the logarithm of the self-rated just income was computed (i.e., ln(y) − ln(y_r_)).

In sensitivity analysis, (1) absolute differences and (2) log differences between factual and self-rated just income (1: y − y_r_; 2: ln(y) − ln(y_r_)) were used as explanatory variables (i.e., without focusing on asymmetric effects).

In further analysis, as some differences between women and men in the association between income comparisons and SWB were reported in the literature [3], fixed effects (FE) regressions were rerun stratified by sex.

As for sociodemographic control variables, age, and family status (married, living together with my spouse; others (married, living (permanently) separated from my spouse; single; divorced; widowed) were used. In addition, the question “Are you legally classified as handicapped or capable of gainful employment only to a reduced extent due to medical reasons?” (no; yes) was used as a surrogate to study changes in morbidity. Furthermore, self-rated health was used (ranging from 1 = “very good” to 5 = “bad”). 

### 2.4. Statistical Analysis

Linear FE regressions were used in the current study as it is very important to control for unobserved heterogeneity (e.g., genetic disposition) in SWB research [14]. FE regressions allow associations between factors constant within individuals over time (e.g., country of origin or sex) and independent variables used in regression analysis [15]. In contrast, random effects (RE) regressions are biased when this association is present [15]. Our choice was also corroborated by Sargan-Hansen tests (results not shown, but available upon request) [16]. The Sargan-Hansen test equals a Hausman test with cluster-robust standard errors. As suggested by Stock and Watson [17], cluster-robust standard errors were computed.

## 3. Results

### 3.1. Descriptive Statistics

Pooled sample characteristics for individuals included in FE regression analysis (with life satisfaction as outcome measure) are depicted in Table 2 (15,942 observations). 

In sum, about one-half were female (47.1%). The mean age was 42.5 (±11.7 years; 17–82 years). More than one out of two (55.4%) were married, living together with spouse. Mean self-rated health was 2.6 (±0.9; 1–5) and 93.5% were not severely disabled. The mean difference between the factual income (y) and self-rated just income was €703.2 (±€1571.1; −€11,000 to €94,139), indicating that on average, factual income was lower than self-rated just income among the individuals. The mean life satisfaction score was 6.8 (±1.7; 0–10).

In addition, the mean positive emotions score was 3.5 (±0.8; 1–5) and the mean negative emotions score was 2.5 (±0.8; 1–5).

### 3.2. Pairwise Correlations

To obtain a first impression of the relationship between the variables of interest, zero-order correlations were computed. These pairwise cross-sectional correlations are presented in Table 3 (for individuals included in FE regression analysis). 

While the variable “richer” (worth repeating: Factual income > just income) was not associated with the outcome measures (negative emotions, positive emotions, and life satisfaction), the variable poorer (factual income < just income) was associated with negative emotions (*r* = 0.03, *p* < 0.01) and life satisfaction (*r* = −0.06, *p* < 0.001). Please see Table 3 for further details.

### 3.3. Regression Analysis

Results of linear FE regression analysis are presented in Table 4. In column 1, negative emotions were used as outcome measure, in column 2, positive emotions were used as outcome measure, and in column 3, life satisfaction was used as outcome measure. 

FE regressions revealed that positive emotions increased with positive income comparisons in the total sample (*β* = 0.16, *p* < 0.05) longitudinally. In contrast, negative income comparisons affect neither negative emotions nor satisfaction with life longitudinally. 

Regarding the control variables, both, age and marital status were associated with negative emotions (age: *β* = 0.02, *p* < 0.001; married, living together with spouse: *β* = −0.12, *p* < 0.05) and life satisfaction (age: *β* = 0.02, *p* < 0.001; married, living together with spouse: *β* = 0.17, *p* < 0.05). While self-rated health was associated with all outcome measures (negative emotions: *β* = 0.19, *p* < 0.001; positive emotions: *β* = −0.18, *p* < 0.001; life satisfaction: *β* = −0.42, *p* < 0.001), disability was not associated with any of the outcome measures.

In sensitivity analysis (results not shown, but available upon request), we also tested whether (i) absolute differences as well as (ii) log differences between factual and self-rated just income affect our outcome measures. However, neither absolute nor log income differences affected the outcome measures in the total sample (and in both sexes).

In further analysis, FE regressions were rerun stratified by sex (results not shown, but available upon request). Longitudinally, positive emotions increased with positive income comparisons in women (*β* = 0.33, *p* < 0.05), but not in men.

## 4. Discussion

Based on a nationally representative longitudinal study of German Households, the aim of this study was to examine the impact of intraindividual income comparisons on subjective well-being (covering AWB and CWB) using FE regression analysis. Regression analysis revealed that longitudinally, positive emotions increased with positive income comparisons in the total sample and in women, but not in men. In sum, these results suggest that comparisons are asymmetric. In contrast, negative income comparisons did not affect SWB.

In previous literature, it was often found that satisfaction with life is lower when an individual’s income is lower than the income of a reference group (interindividual) [3,18]. Moreover, an experimental study showed that expectations (expectations about the current round’s payment) negatively affect satisfaction [19]. Another study [20] investigated whether “major improvement in financial situation (e.g., won lottery, received an inheritance)” or “major worsening in financial situation (e.g., went bankrupt)” affect life satisfaction in the long run. Using Household, Income and Labour Dynamics in Australia (HILDA) data, they showed that a negative financial event has a markedly greater effect on life satisfaction than a positive financial event. However, people tend to adapt (incompletely) after two years to both events. Furthermore, Frijters et al. [21] investigated whether an expected change in household income in the next five years affects life satisfaction in China. They found that optimistic expectations are associated with higher life satisfaction. However, while other studies analyzed interindividual income comparisons or investigated income expectations (past and future income expectations), we examined the distance between own individual income and fair income. As mentioned in the introduction, only one study showed an association between perceived income injustice and stress-associated diseases [6].

Quite surprisingly, we found that positive emotions increased with positive income comparisons in women (but not in men), whereas no association with negative income comparisons was found in women and in men. Given the previous findings concerning interindividual income comparisons, it appears plausible that men are not affected by positive intraindividual income comparisons. However, it is quite puzzling that men are not affected by negative intraindividual income comparisons. A possible, but speculative, explanation might be that men only attach great importance to external income comparisons for status reasons. Thus, it appears plausible that men are more status-oriented and competitive. In contrast, women might have lower expectations [22]. However, future research is required to test these speculative assumptions. Another explanation may be that underlying personality factors [23] affect the link between intraindividual income comparisons and well-being. Additionally, gender roles [24] may play a role in this link. 

The current study has some strengths. Our study provides the first insights into the relation between intraindividual income comparisons and SWB. In addition, asymmetric comparison effects were examined. By using FE regressions, insights into the causal relationship can be given (with certain restrictions since no controlled stimulus exists). Moreover, time-constant factors (observed and unobserved) can be taken into consideration. Additionally, data were used from a large, population-based longitudinal study (GSOEP). However, some caveats are worth highlighting. Individuals rating their earning as just, might earn an income that is above their perception of a just income. These are individuals that replied to the question “Is the income that you earn at your current job just, from your point of view” with yes. These individuals did not answer the question regarding a just income. Consequently, estimations might be slightly biased. As this is a longitudinal study, panel attrition might occur. However, it has been demonstrated that the GSOEP has very low attrition rates [10].

## 5. Conclusions

Our findings stress the importance of positive intraindividual income comparisons for positive emotions. For example, strategies to shift income expectations (for example, embedded in cognitive-behavioral psychotherapy or by restructuring life goals) might be beneficial for increasing positive emotions.

With regard to future research, it appears plausible that several factors (e.g., personality factors [24]) may act as moderating factors in this link. Thus, we recommend future research in this area. 

## Figures and Tables

**Table 1:** Asymmetric effects (variables “poorer” and “richer”).

Scenario	Variables: Richer/Poorer
If y > y_r_, then…	Richer = ln(y) − ln(y_r_)
	Poorer = 0
If y < y_r_, then…	Richer = 0
	Poorer = ln(y_r_) − ln(y)
If y = y_r_, then…	Richer = 0
	Poorer = 0

**Table 2:** Sample Characteristics for individuals included in fixed effects regressions (Wave 2005, 2007, 2009, 2011 and 2013, pooled; 15,942 observations).

Variables	*N* (%)/Mean (SD); Range
Female: *N* (%)	7512 (47.4%)
Age (in years): Mean (SD)	42.5 (11.7); 17–82
Married, living together with spouse: *N* (%)	8839 (55.0%)
Self-rated health (from 1 = “very good” to 5 = “bad”)	2.6 (0.9); 1–5
Not severely disabled: *N* (%)	14,898 (93.4%)
Life satisfaction: Mean (SD); Range	6.8 (1.7); 0–10
Difference: Self-rated just income–factual income in €	703.2 (1571.1); −11,000–94,139
Life satisfaction (from 0 = “completely dissatisfied” to 10 = “completely satisfied”).	6.8 (±1.7; 0–10)

Comments: The explanatory variable sex was not included in FE regressions as an independent variable because it is time-invariant (it usually did not vary within individuals over time). It was used for descriptive purposes.

**Table 3:** Pairwise cross-sectional correlations (with Bonferroni-adjusted significance level; Wave 2005, 2007, 2009, 2011 and 2013, pooled; 15,942 observations).

Variables	Age in Years	Married, Living Together with Spouse (Ref.: Other)	Severely Disabled (Ref.: Not Severely Disabled)	Self-Rated Health (from 1 = “Very Good” to 5 = “bad”)	Richer	Poorer	Negative Emotions	Positive Emotions	Life Satisfaction
Age in years	1.00								
Married, living together with spouse (Ref.: Other)	0.39 ***	1.00							
Severely disabled (Ref.: Not severely disabled)	0.16 ***	0.02	1.00						
Self-rated health (from 1 = “very good” to 5 = “bad”)	0.25 ***	0.08 ***	0.22 ***	1.00					
Richer	−0.00	0.00	−0.01	0.01	1.00				
Poorer	0.02	0.02	0.02 ^†^	0.01	−0.06 ***	1.00			
Negative emotions	−0.05 ***	−0.06 ***	0.05 ***	0.31 ***	0.00	0.03 **	1.00		
Positive emotions	−0.16 ***	0.03 **	−0.06 ***	−0.30 ***	−0.01	−0.02	−0.31 ***	1.00	
Life satisfaction	−0.08 ***	0.04 ***	−0.09 ***	−0.40 ***	0.02	−0.06 ***	−0.41 ***	0.45 ***	1.00
Observations	15,942								

*** *p* < 0.001, ** *p* < 0.01, * *p* < 0.05, ^†^ *p* < 0.10. In the case of life satisfaction, the waves 2005, 2007, 2009, 2011 and 2013 were used. In the case of negative emotions or positive emotions, the waves 2007, 2009, 2011 and 2013 were used. Negative emotions range from 1 = rare negative emotions to 5 = frequent negative emotions; Positive emotions range from 1 = rare positive emotions to 5 = frequent positive emotions; Life satisfaction range from 0 = “completely dissatisfied” to 10 = “completely satisfied”; Individuals were classified as “richer“ (“poorer”) if their factual income was higher (lower) than their self-rated just income. Please see the Methods section (subsection ‘Intraindividual income comparisons’) for further details.

**Table 4:** Determinants of subjective well-being (column 1: Negative emotions as outcome measure; column 2: Positive emotions as outcome measure; column 3: Life satisfaction as outcome measure). Results of linear FE regressions (Wave 2005, 2007, 2009, 2011 and 2013).

Independent Variables	Negative Emotions	Positive Emotions	Life Satisfaction
Age	−0.0163 ***	0.00262	0.0231 ***
	(0.00357)	(0.00401)	(0.00586)
Married, living together with spouse (Ref.: Other ((married, living (permanently) separated from my spouse; single; divorced; widowed)	−0.115 *	0.0675	0.174 *
	(0.0453)	(0.0518)	(0.0764)
Severely disabled (Ref.: Not severely disabled)	0.0721	−0.0981	−0.129
	(0.0646)	(0.0774)	(0.128)
Self-rated health (from 1 = “very good” to 5 = “bad”)	0.185 ***	−0.180 ***	−0.424 ***
	(0.0148)	(0.0167)	(0.0272)
Richer	0.0888	0.157 *	−0.0444
	(0.0670)	(0.0721)	(0.0948)
Poorer	0.00291	−0.0182	−0.0916
	(0.0337)	(0.0406)	(0.0658)
Constant	2.759 ***	3.823 ***	6.820 ***
	(0.153)	(0.175)	(0.253)
Observations	13,430	13,423	15,942
R^2^	0.044	0.030	0.049
Number of Individuals	8425	8422	9259

Beta-coefficients are reported; Cluster-robust standard errors in parentheses; *** *p* < 0.001, ** *p* < 0.01, * *p* < 0.05, ^†^ *p* < 0.10. In the case of life satisfaction, the waves 2005, 2007, 2009, 2011 and 2013 were used. In the case of negative emotions or positive emotions, the waves 2007, 2009, 2011 and 2013 were used. Negative emotions range from 1 = rare negative emotions to 5 = frequent negative emotions; Positive emotions range from 1 = rare positive emotions to 5 = frequent positive emotions; Life satisfaction range from 0 = “completely dissatisfied” to 10 = “completely satisfied”; Individuals were classified as “richer” (“poorer”) if their factual income was higher (lower) than their self-rated just income. Please see the methods section (subsection ‘Intraindividual income comparisons’) for further details.
